# Mode and Frame Matter: Assessing the Impact of Survey Mode and Sample Frame in Choice Experiments

**DOI:** 10.1177/0272989X19871035

**Published:** 2019-09-15

**Authors:** Verity Watson, Terry Porteous, Tim Bolt, Mandy Ryan

**Affiliations:** Health Economics Research Unit, University of Aberdeen, Foresterhill, Aberdeen, Scotland; Health Economics Research Unit, University of Aberdeen, Foresterhill, Aberdeen, Scotland; Faculty of Economics, Saitama University, Sakura-ku, Saitama, Japan; Health Economics Research Unit, University of Aberdeen, Foresterhill, Aberdeen, Scotland

**Keywords:** choice experiment, health care, sample frame, survey mode

## Abstract

**Background.** Choice experiments (CE) are applied in health economics to elicit public preferences and willingness to pay (WTP). CEs are frequently administered as Internet-based surveys. Internet surveys have recognized advantages, but concerns exist about the representativeness of Internet samples, data quality, and the impact on elicited values. **Aim.** We conducted the first study in health comparing an Internet-based CE survey with the more traditional general population mail survey. We also compared the Internet-based and mail CE surveys with computer-assisted personal interviews (CAPIs), which are commonly used to elicit health state valuations. **Methods.** Two separate samples were drawn from 2 United Kingdom (UK) volunteer Internet panels (IPs), CAPIs were undertaken with respondents sampled from UK Census Output Areas, and mail surveys were sent to UK households drawn from the postcode address file (PAF). Each mode received more than 1000 respondents. We compared modes and frames using objective measures (response rate, sample representativeness of the UK population, elicited values, theoretical validity, and cost per response) and subjective/self-reported measures (time taken to complete the study, perceived study consequentiality, and stated attribute nonattendance). This study intentionally confounded the survey modes and sample frame by choosing sample frames that are typically used by researchers for each mode. **Results.** Estimated WTP differs across mode-frame pairs. On most measures, CAPIs dominated. They are more expensive, however. On all measures, except response rates, Internet surveys dominated the mail survey. They were also cheaper. **Conclusion.** Researchers using IPs should pay attention to response rates and be aware that the quality of IPs differs. Given the importance of perceived consequentiality and attribute attendance in CEs, future research should address their impact across modes and frames.

Health economists frequently use surveys to collect data, commonly using postal questionnaires and in-person interviews. More recently, the Internet has been used to administer surveys as a potential solution to falling response rates to postal surveys. Internet surveys also offer several advantages over postal surveys: lower data collection costs, increased data collection speed, the possibility of including multimedia elements, and automatic data entry.^1^ Internet surveys also offer a less expensive alternative to in-person interviews commonly used for health state valuation studies. The survey mode may, however, influence who is asked to respond to a survey (sample frame), who does or does not respond to a survey (nonresponse bias), how respondents answer the survey questions, and the respondents’ ability to provide accurate responses (measurement error).

Literature on the effects of survey mode in health economics is limited in the number of studies and the modes compared. The literature also provides mixed evidence of survey mode effects. Norman et al.^2^ compared a computer-assisted personal interview (CAPI) and online survey to elicit health state valuations using a time-tradeoff task. They found the online survey had higher variability and more extreme responses than in-person interview valuations.^2^ In contrast, Mulhern et al.^3^ compared a CAPI survey and Internet panel (IP) survey to elicit health state valuations using a choice experiment (CE) and found no difference in valuations across modes but found significant differences in respondents’ characteristics. They concluded that both modes may be equally valid. Rowen et al.^4^ compared online and face-to-face interviews in a pairwise comparison study of social preferences for burden of illness. They found that the mode of administration affected responses and the socioeconomic characteristics of respondents. Determann et al.^5^ compared patient preferences for health insurance elicited using a CE across 2 samples drawn from an IP’s membership: one sample completed the questionnaire online, and the other received a mail survey. They found no evidence that online surveys yield inferior results compared with paper-based surveys but that they did have a lower price per completed respondent.

In-person interviews have been the mode of choice for health state valuation studies and for studies eliciting social preferences. Therefore, a comparison between in-person and online surveys is particularly relevant to the contexts studied by Mulhearn et al.^3^ and Rowen et al.^4^ Between 2000 and 2012, about half of all CE studies eliciting preferences for health and care were mail surveys, and 6% were online surveys,^6^ but since 2013, more than half have been administered via the Internet.^7^ No study has tested whether the results of a CE survey of health care preferences differ when the survey mode is a mail survey of the general population or an IP survey. Although Determann et al.^5^ compared an online survey with pen-and-paper completion, both samples were drawn from the same IP.

We present the first study comparing CE results across IP surveys and a mail survey sent to UK households from the postcode address file (PAF) and a CAPI of UK households. Given differences across the operation and composition of IPs, we compared responses for 2 IPs. We compared modes and the sample frames most commonly associated with them using objective measures (response rate, representativeness of the samples [compared with the UK population], elicited values, theoretical validity, cost per response) and subjective/self-reported measures (time taken to complete the survey, perceived study consequentiality, stated attribute nonattendance).

## Experimental Design

### The CE

**Figure 1:**
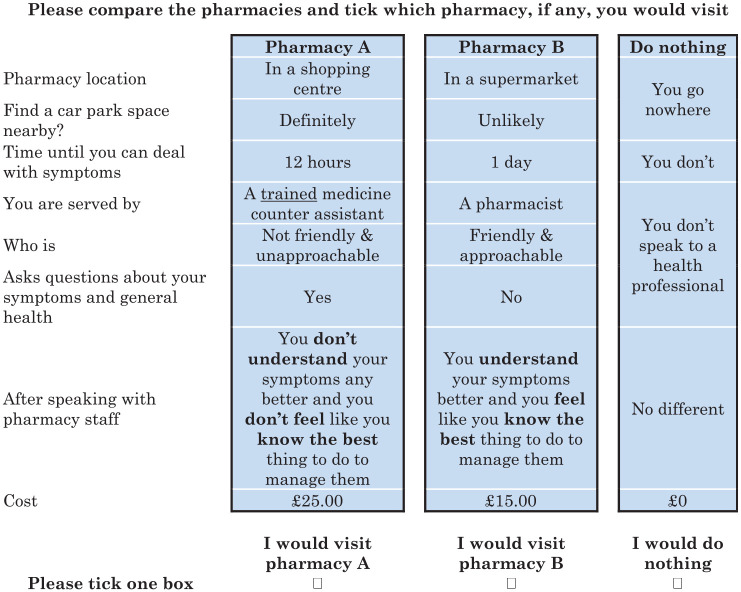
Example of choice set included in the choice experiment.

The CE elicited general population preferences for characteristics of community pharmacies when managing a minor ailment (cold or flu).^8^ This is a health care “good” relevant to the general population. The attributes and levels (Table 1) were selected based on a review of quantitative and qualitative studies that elicited preferences for the use of pharmacy services to manage minor ailments and a parallel cohort study investigating how the public used pharmacy services to manage minor ailments.^8^ Respondents were asked to complete a series of choice tasks in which they had to choose among 3 options: 2 pharmacy service options and doing nothing. In the minor ailment context, doing nothing is a realistic option. The experimental design, selected using SAS v9.2 and the Mktex macro,^9^ resulted in 48 choice tasks. To reduce the burden to each respondent and maximize response rates, we blocked these into 6 blocks of 8 choice sets. An example choice set is presented in Figure 1.

**Table 1:** Attributes and Levels Included in the Choice Experiment

Attribute	Levels
Pharmacy location	At the local shops In a shopping center In a supermarket Beside a doctor’s surgery
Will you find a car parking space nearby?	No Unlikely Probably Definitely
Time until you can deal with your symptoms	5 hours 12 hours 1 day 2 days
You are served by	A pharmacist A trained medicine counter assistant An untrained medicine counter assistant
Who is	Not friendly and unapproachable Friendly and approachable
Are you asked questions about your symptoms and your general health	Yes No
After speaking to the pharmacy staff	You understand your symptoms better and you feel like you know the best thing to do to manage them You don’t understand your symptoms better and you don’t feel like you know the best thing to do to manage them
Cost	£2.50, £7.50, £15.00, £25.00

The questionnaire also collected information on respondents’ socioeconomic characteristics, health status, attitudes and beliefs (both about health care and in general), and data quality. The questionnaire is available online at the UK data service.^10^

### Modes of Data Collection and Sample Frames

We compared 3 survey modes: CAPI, mail, and IP. Data collection was concurrent across modes. Given IP surveys are quicker than both CAPI and mail surveys, data collection for this mode was staggered over 3 weeks. We aimed to receive 150 responses per mode in the pilot and 1000 responses per mode in the main study. We used a unimode approach, with the same questionnaire used across all modes to ensure survey differences across modes were minimal.^1^ The IP and CAPI surveys were computer screen based, and the mail survey was a pen-and-paper, self-completed questionnaire.

We applied each method of data collection as it is typically applied by researchers; thus, for each mode, we used the most common UK sample frame to create a mode-frame pair:

- The mail survey was sent to a random sample of UK residential postal addresses from the PAF, stratified by geographical region. For the pilot study 1000 questionnaires and for the main study 6669 questionnaires were mailed out, based on an expected response rate of 15%. Each envelope was addressed to “the occupier,” and the invitation requested that “the person in the household, of 18 years or over, whose birthday fell next” should complete the questionnaire. Nonrespondents received a reminder and duplicate questionnaire 3 weeks after the initial mailing. Ipsos-MORI administered the mail survey.
- For the IP mode, samples were drawn from 2 UK volunteer IPs: Ipsos-MORI (IP-IM) and ResearchNow (IP-RN). These panels differ in member recruitment and reward, and the management of inattentive or dishonest members.^11^ Such differences may affect the sample available to survey, the characteristics of respondents, and respondents’ task engagement. The IP sample frames were stratified by age, gender, working status and geographical location. Potential participants were selected at random from those eligible within strata. Invitations to complete the questionnaire were emailed to the sample, with 2 reminders to nonrespondents. When individuals responded to the invitation, they were screened against the quotas until the target number of responses had been received. As per standard practice, respondents received a nominal incentive in the form normally used by the survey companies as a reward for completing the survey.
- The CAPI sample frame was based on UK Census Output Areas (COAs), stratified by region. Trained interviewers recruited participants in their own homes; invitations were issued verbally by interviewers to potential respondents. Approaches within a COA were guided by quotas (age, gender, and working status); interviewers worked to these quotas until the target number of responses had been achieved. The CAPI data were collected by Ipsos-MORI.

## Comparison Tests, Hypotheses, and Results

We compared 4 data sets (CAPI, mail, IP-IM, IP-RN) according to response rates, respondent representativeness, values elicited, data quality (theoretical validity, time to complete survey, perceived consequentiality, and attribute nonattendance), and cost per response. Our results are summarized in Table 2.

**Table 2:** Summary of Results across Mode-Frame Pairs

	CAPI	Mail	IP-IM	IP-RN
Response/participation rate, %	27.0	17.2	12.0	4.1
Cost per respondent, £	37.50	18.20	8.20	2.50
Sociodemographic representativeness of UK population	Yes, for gender, education, Internet access	No	Yes, for gender	Yes, for gender and voting attitude
Differences in characteristics across mode-frame pairs	Chronic illness and MAS member differences v. mail, IP-IM, P-RM BMQ difference v. mail, IP-RN	Chronic illness difference v. CAPI, IP-IM, IP-RN MAS difference v. CAPI BMQ differences v. CAPI, IP-IM	Chronic illness difference v. CAPI, mail MAS difference v. CAPI BMQ differences v. mail, IP-RN	Chronic illness difference v. CAPI, mail MAS difference v. CAPI BMQ differences v. CAPI, mail, IP-IM
Elicited values (mWTP)^2^	Differ across ALL mode-frame pairs Generally lower than mail and higher than IP-IM and IP-RN	Differ across ALL mode-frame pairs Higher than other mode-frame pairs	Differ across all mode-frame pairs Lower than other mode-frame pairs	Differ across all mode-frame pairs Lower than CAPI and mail, higher than IP-IM
Data quality				
Theoretical validity	Yes	Yes	Not monotonic across “car parking” attribute levels	Yes
Survey completion time, min, mean (SD)	21.7 (6.27)	21.3 (9.5)	15.8 (8.3)	15.7 (8.1)
Perceived consequentiality	21.5% strongly/disagree 26.7% uncertain 51.8% strongly/agree	17.4% strongly/disagree 36.8% uncertain 39.8% strongly/agree	16.4% strongly/disagree 35.1% uncertain 48.5% strongly/agree	13.5% strongly/disagree 31.6% uncertain 54.9% strongly/agree
ANA, all attributes, and costs	64.4% attend all attributes 8.4% ignore cost	51.3% attend all attributes 9.9% ignore cost	74.5% attend all attributes 2.6% ignore cost	73.5% attend all attributes 3.1% ignore cost

ANA, attribute nonattendance; BMQ, Beliefs about Medicines Questionnaire; CAPI, computer-assisted personal interviews; IP-IM, Internet panel Ipsos-MORI; IP-RN, Internet panel ResearchNow; mail, postal; MAS, minor ailments scheme; mWTP, marginal willingness to pay.

### Response Rates

We used the American Association for Public Opinion Research guidelines for response (participation) rate calculations.^11^ Given it is not possible to calculate response rates for volunteer IP panels, we calculated participation rates. The results are shown in Table 3. CAPI had the highest rate and IP-RN the lowest. Participation rates for both IPs were lower than response rates for both offline mode-frame pairs and differed across IPs.

**Table 3:** Response and Participation Rates across Survey Mode-Frame Pairs, No.

	CAPI	Mail	IP-IM	IP-RN
Total administered	14006	6669	8881	26078
Total completed	1049	1122	1000	1000
Unavailable/ineligible^a^	10121	150	550	1473
Nonresponse^b^	2836	5397	7331	23605
Response/participation rate, %	27	17	12	4

CAPI, computer-assisted personal interviews; IP-IM, Internet panel Ipsos-MORI; IP-RN, Internet panel ResearchNow; mail, postal.

a Respondents were unavailable in the CAPI if no one answered the door and in the mail if the questionnaire was undeliverable. Respondents were ineligible in the CAPI if they fell into a quota category in which the quota had already been achieved. Respondents were ineligible in the Internet surveys if they were younger than 18 years or if they fell into a quota category in which the quota had already been achieved. No one was ineligible in the mail mode.

b Nonresponse in the mail survey means that the survey was not returned; in the CAPI, this means that respondents answered the door but refused to take part in the survey, and in the Internet panels, this means that respondents received the e-mail invitation but did not start or complete the survey.

### Respondent Representativeness

Survey response rates are linked to nonresponse error, which occurs when individuals who respond to the survey differ from those who do not respond. We compared respondent characteristics with the population to understand if there are observable differences between the samples that result from responses to a mode-frame pair. We note that unobservable differences may remain.

We compared each survey mode-frame pair’s respondents with the UK general population, based on a broad set of socioeconomic and health characteristics (see Table 4). We supplemented this with 2 additional characteristics—Internet access and voting attitude*—*as a proxy for social attitudes. All IP respondents had Internet access, but we collected Internet access data in the CAPI and mail surveys to gauge the number of respondents who do not have Internet access and therefore would be restricted in accessing IPs.

**Table 4:** Differences between Respondents’ Characteristics and the UK Population by Survey Mode-Frame Pairs

Variable	Variable Definition	Test Applied	Survey Mode-Frame Pairs, *P* Value			
			CAPI	Mail	IP-IM	IP-RN
Age	5-year intervals	χ^2^	0.003	<0.001	<0.001	<0.001
Gender	% male	Binomial	1.000	<0.001	0.974	0.681
Education	% degree or higher	Binomial	0.102	<0.001	<0.001	<0.001
Employment	8 categories	χ^2^	<0.001	<0.001	<0.001	<0.001
Self-assessed health	5 categories	χ^2^	<0.001	<0.001	<0.001	<0.001
Activities of daily living	% limited	χ^2^	0.013	<0.001	<0.001	<0.001
Income	9 income bands	χ^2^	<0.001	<0.001	<0.001	<0.001
Internet access	% with access	binomial	0.110	<0.001	<0.001	<0.001
Voting attitude	3 categories	χ^2^	0.015	<0.001	0.005	0.065

As 9 tests are applied to each mode-frame pair for sample representativeness, to reduce the risk of type 1 errors, we applied a Bonferroni correction and used 0.0055 as the significance level to maintain a family-wise error rate of 0.05. Actual values and how the test variables are constructed can be found in Supplementary Appendix A, Tables A2 and A3, which report the respondent characteristics for each of the mode-frame pairs. CAPI, computer-assisted personal interviews; IP-IM, Internet panel Ipsos-MORI; IP-RN, Internet panel ResearchNow; mail, postal.

We used population data from 4 sources: the 2011 UK census (age, gender, education, employment, self-assessed health, activities of daily living^12^), the Family Resources Survey (income^13^), Eurostat community survey on Information and Communication Technologies (ICT) usage in households and by individuals (Internet access^14^), and British Social Attitudes Survey (voting attitude^15^). We hypothesized that CAPI respondents would be older and more likely to be retired than the general population^4^ and that IP respondents would have higher educational qualifications,^2^ have lower health status, be younger, and be less likely to be employed or retired.^4^

We also compared respondents across mode-frame pairs based on 3 additional health-related variables for which there is no UK population data: experience of chronic illness, membership of a minor ailments scheme (MAS), and general beliefs about medicines. These characteristics are likely to influence routine use of community pharmacies, which may in turn affect CE responses and subsequent engagement/quality of data. Individuals who experience a chronic illness may be more likely to attend a pharmacy than those with no such illness. We hypothesized that IP respondents would be more likely to have a chronic illness than CAPI respondents.^4^ MASs allow eligible National Health Service patients in the UK (e.g., those in receipt of social benefits or exempt from paying prescription charges) to receive free advice and medicines from community pharmacies for specific minor ailments (https://www.gov.scot/publications/nhs-minor-ailment-service-local-pharmacy/); MAS membership signals that respondents already attend the pharmacy for minor ailments. The Beliefs about Medicines Questionnaire (BMQ) is a validated instrument developed to better understand people’s perceptions about medicines.^16^ Previous studies have found that an individual’s beliefs about medicines can influence health behavior, particularly their medicine use.^17–20^ We used the BMQ-General, a measure of the extent to which respondents believe that medicines in general are overused, harmful, or beneficial (see Supplementary Appendix, Table A1). We made no a priori hypotheses regarding membership of MAS and responses to the BMQ.

Respondents’ characteristics across survey mode-frame pairs are reported in the Supplementary Appendix (Tables A2 and A3). Statistical comparison tests are presented in Table 4. Compared with the UK population:

- CAPI respondents were older, overrepresenting the population in all age categories from 65 to 84 years, less likely to be an employee and more likely to have retired, to be in worse health, and earning less than the UK population.
- Mail respondents were older (in particular, respondents overrepresented the population in all categories older than 54 years), more likely to be female, better educated, more likely to be retired, in worse health, more likely to have a lower income, less likely to have Internet access, and more likely to state that it was one’s duty to vote than the UK population.
- IP respondents were overrepresented by people aged 30 to 34 years and 65 to 74 years, better educated, more likely to be retired, in worse health, overrepresented by people with low or high incomes, and more likely to have Internet access than the UK population.

The distribution of the 3 additional health-related characteristics are reported in the Supplementary Appendix, Table A4. Table 5 reports comparison tests for these characteristics. Mail and CAPI respondents were more likely to report a chronic illness than IP respondents, and mail respondents were more likely to report a chronic illness than CAPI respondents. CAPI respondents were twice as likely to be MAS members as respondents to other mode-frame pairs. CAPI respondents were more likely than mail and IP-IM respondents to believe medicines are overused, and CAPI and IP-IM respondents were more likely to believe medicines are harmful than were mail or IP-RN respondents.

**Table 5:** Differences between Respondents’ Health Characteristics and Attitudes across Survey Mode-Frame Pairs

	Survey Mode-Frame Pair, *P* Value			
		CAPI	Mail	IP-IM
	Mail	<0.001	N/A	
Chronic illness	IP-IM	<0.001	<0.001	N/A
	IP-RN	<0.001	<0.001	Matched^a^
	Mail	<0.001	N/A	
Member of minor ailments scheme	IP-IM	<0.001	0.857	N/A
	IP-RN	<0.001	0.211	0.407
Beliefs about Medicines Questionnaire				
	Mail	0.002	N/A	
Overuse	IP-IM	0.190	0.089	N/A
	IP-RN	0.014	0.491	0.299
	Mail	<0.001	N/A	
Harm	IP-IM	0.117	<0.001	N/A
	IP-RN	<0.001	0.037	0.004
	Mail	0.718	N/A	
Benefit	IP-IM	0.379	0.577	N/A
	IP-RN	0.658	0.920	0.660

CAPI, computer-assisted personal interviews; IP-IM, Internet panel Ipsos-MORI; IP-RN, Internet panel ResearchNow; mail, postal.

a All (100%) respondents to IP-IM and IP-RN reported “no” chronic illness.

### Elicited Values

We estimated respondents’ preferences for pharmacy service attributes using an error components logit model. We assumed that the utility *u* of individual *i* for alternative *j* in choice set *t* for mode *m* is based on a systematic component specified as a linear and additive function of the study attributes and levels and an additive random component $\varepsilon_{\mathrm{ijt}}$ . The error-components model allowed us to merge data across all mode-frame pairs, accounting for mode-frame–specific scale differences. We thus estimated

(1) $$U_{\mathrm{ijtm}}=d_{m}\times \left(\alpha_{\mathrm{no}}+\alpha_{B}+\sum_{n=1}^{N}\beta_{n}x_{n}\right)+\theta z_{i}d_{m}+\varepsilon_{\mathrm{ijt}}$$

where *$d_{m}$* is a dummy variable (that takes the value “1” if an alternative appears in mode *m* and “0” otherwise), $\alpha_{\mathrm{no}}$ and $\alpha_{B}$ are alternative specific constants that control for aspects other than pharmacy characteristics (these explain respondents’ choice of using a pharmacy service rather than taking no action ( $\alpha_{\mathrm{no}}$ ) and any left/right bias in respondents’ choice of pharmacy ( $\alpha_{B}$ )), $x_{n}$ are the levels of the n=1…N attributes included in the experimental design, $\beta_{n}$ are their respective parameter estimates, θ represents a deviation from the mean alternative specific constant, and *$z_{i}$* is a draw from a normal distribution N(0,1). We estimated separate error components for each mode-frame pair. We specified an additive error term $\varepsilon_{\mathrm{ijt}}$ that is Gumbel distributed.

Following estimation of Equation 1, we explored differences in the estimated mean marginal willingness to pay (mWTP) across mode-frame pairs. We calculated the difference in mWTP for pairs of mode-frame pairs and tested if these differences were significant using the delta method.^21^ We hypothesized no differences in mWTP across respondents.

We found differences across mode-frame pairs (Table 6). CAPI and mail, and IP-IM and IP-RN were most similar. Mail respondents’ mWTP estimates were significantly different from other mode-frame pairs for all attributes except “pharmacy location”; they were the highest across all mode-frame pairs and for some attributes they were twice as large. CAPI and both IPs revealed significant differences in mWTP for “pharmacy location,”“who you speak to,” whether they are “friendly,” and whether you “better understand symptoms.” mWTP estimates across the IPs were significantly different for “who you speak to,” whether they are “friendly,” and whether you “better understand symptoms.” mWTP estimates were lowest among IP respondents.

**Table 6:** Comparing Marginal Willingness-to-Pay (mWTP) Estimates across Survey Mode-Frame Pairs

	CAPI mWTP	Mail mWTP	IP-IM mWTP	IP-RN mWTP	CAPI/Mail Ratio	CAPI/IP-IM Ratio	CAPI/IP-RN Ratio	Mail/IP-IM Ratio	Mail/IP-RN Ratio	IP-IM/IP-RN Ratio
Shopping center^a^	−£5.05	−£3.56	−£2.05	−£2.33	1.42	2.46***	2.17**	1.73	1.53	0.88
Supermarket^a^	−£3.25	−£2.24	−£1.22	−£1.52	1.45	2.66*	2.14	1.83	1.47	0.80
Doctor’s surgery^a^	ns	ns	ns	ns	/	/	/	/	/	/
Car park, probably^b^	−£1.96	−£3.38	−£1.58	−£2.72	0.57	1.24	0.71	2.14	1.24	0.58
Car park, unlikely^b^	−£3.71	−£5.96	−£4.01	−£3.75	0.62*	0.93	0.99	1.49*	1.59**	1.07
Car park, no^b^	−£3.97	−£6.40	−£3.86	−£4.63	0.62*	1.03	0.86	1.64**	1.38	0.83
Time (per hour)	−£0.23	−£0.32	−£0.21	−£0.20	0.72***	1.09	1.15	1.52***	1.60***	1.05
Trained MCA^c^	ns	ns	ns	ns	—	—	—	—	—	—
Untrained MCA^c^	−£8.64	−£11.85	−£5.84	−£9.03	0.73***	1.48***	0.96	2.03***	1.31***	0.65***
Friendly^d^	£6.93	£6.91	£3.55	£4.61	1.01**	1.95***	1.50***	1.95***	1.50***	0.77**
Asked questions^e^	£3.15	£3.70	£2.59	£2.75	0.85	1.22	1.15	1.43*	1.35	0.94
Understand symptoms^f^	£12.46	£16.87	£8.07	£10.27	0.74***	1.54***	1.21***	2.09***	1.64***	0.79***
Average ratio					0.87	1.56	1.28	1.79	1.46	0.83

Results are unchanged when the model is estimated with a mixed logit model with normal distributions for all attributes expect cost. CAPI, computer-assisted personal interviews; IP-IM, Internet panel Ipsos-MORI; IP-RN, Internet panel ResearchNow; mail, postal; MCA=Medicines Counter Assistant, ; ns=not significant, . Asterisks indicate statistically significant differences at the *10%, **5%, and ***1% level, respectively.

a Compared with “local shops.”

b Compared with “car park, definitely.”

c Compared with “pharmacist.”

d Compared with “unfriendly.”

e Compared with “not asked questions.”

f Compared with “do not understand symptoms.”

### Data Quality

#### Theoretical validity

Across mode-frame pairs, we compared if the parameters estimated with the error-component model were in line with a priori hypotheses. We hypothesized that respondents would prefer being able to park, shorter waiting times for treatment, being seen by a more qualified person who is friendly and approachable, gaining a better understanding of their symptoms, and lower-cost treatments. We had no a priori hypotheses regarding preferences for location or being asked questions about symptoms and general health. We noted which parameters were not in line with a priori assumptions. We hypothesized that the CAPI would have the highest number of parameters in line with assumptions because the interviewer can assist respondents who find the task difficult.

The regression coefficients, reported in Table 7, are in line with a priori hypotheses for all attributes and mode-frame pairs except for the car parking attribute in the IP-IM (where the estimated coefficients are not monotonic across the attribute levels).

**Table 7:** Choice Experiment Regression Results: Testing Theoretical Validity

Attribute	Level	CAPICoefficient (SE)	MailCoefficient (SE)	IP-IMCoefficient (SE)	IP-RNCoefficient (SE)
Constant	No action	−2.081* (0.182)	−2.219* (0.204)	−1.550* (0.170)	−1.987* (0.175)
	Alternative A	0.042 (0.039)	−0.019 (0.043)	0.045 (0.046)	0.078 (0.044)
Location^a^	Shopping center	−0.306* (0.058)	−0.255* (0.065)	−0.225* (0.068)	−0.219* (0.065)
	Supermarket	−0.197* (0.057)	−0.160* (0.065)	−0.133 (0.068)	−0.143* (0.065)
	Doctor’s surgery	0.016 (0.057)	−0.057 (0.064)	−0.101 (0.067)	−0.076 (0.064)
Car parking^b^	Probably	−0.118* (0.060)	−0.242* (0.068)	−0.173* (0.070)	−0.255* (0.068)
	Unlikely	−0.225* (0.058)	−0.427* (0.065)	−0.439* (0.069)	−0.352* (0.066)
	No	−0.241* (0.058)	−0.458* (0.067)	−0.422* (0.069)	−0.435* (0.066)
Time (per hour)		−0.014* (0.001)	−0.009* (0.002)	−0.009* (0.002)	−0.004* (0.002)
Served by^c^	Trained MCA	0.016 (0.050)	−0.105 (0.055)	−0.044 (0.058)	−0.099 (0.055)
	Untrained MCA	−0.523* (0.049)	−0.849* (0.056)	−0.639* (0.058)	−0.848* (0.056)
Friendly^d^		0.419* (0.034)	0.495* (0.038)	0.389* (0.040)	0.433* (0.037)
Asked questions^e^		0.190* (0.032)	0.265* (0.036)	0.284* (0.038)	0.257* (0.036)
Understand symptoms^f^		0.754* (0.033)	1.208* (0.039)	0.883* (0.040)	0.964* (0.038)
Cost		−0.061* (0.003)	−0.072* (0.003)	−0.109* (0.003)	−0.094* (0.003)
Error component		3.893* (0.171)			
Error component (mail)			4.416* (0.201)		
Error component (IP-IM)				3.233* (0.130)	
Error component (IP-RN)					3.436* (0.144)
N obs = 97,707; log likelihood = −24,146					

CAPI, computer-assisted personal interviews; IP-IM, Internet panel Ipsos-MORI; IP-RN, Internet panel ResearchNow; mail, postal; MCA=Medicines Counter Assistant,.

a Omitted level “at local shops.”

b Omitted level “definitely.”

c Omitted level “pharmacist.”

d Omitted level “not friendly and approachable.”

e Omitted level “doesn’t ask questions.”

f Omitted level “don’t understand your symptoms better.”

* Significant at the 5% level.

#### Survey completion decision

Social exchange theory applied to surveys assumes that respondents answer a survey when perceived benefits outweigh costs.^1^ Response costs are the time and effort required to complete the survey; benefits are the reward and the feeling of importance respondents (may) receive from taking part. We proxied response costs using time taken to complete the survey. Computer administered surveys (CAPI and IPs) collected this information automatically. Mail survey respondents manually recorded the time at the beginning and end of the survey. We compared time taken across pairs of mode-frame pairs using a 2-sample *t* test with unequal variances. We hypothesized that IP respondents would complete the survey faster than CAPI or mail respondents.^3^

One proxy for respondents’ benefit of responding is perceived consequentiality of the survey. Carson and Groves^22^ argued that when respondents perceive that their responses are consequential, they have an incentive to reveal their true preferences. Vossler and Watson^23^ found that survey respondents who perceived their responses to be consequential accurately predict votes in a public referendum. We measured what respondents perceived to be the impact of their responses using questions proposed by Scheufele and Bennett.^24^ Respondents were asked whether they thought their answers to the survey would change how services are provided.^24^ Responses were compared across mode-frame pairs using a chi-squared test. No previous studies have compared perceived consequentiality across mode-frame pairs. We hypothesized that CAPI respondents would have the highest perceived consequentiality because the presence of a trained interviewer should signal the importance of the survey.

Table 8 reports the mean and standard deviation of survey completion times and the perceived consequentiality of responses across mode-frame pairs. We found no statistically significant difference in response time between the CAPI and mail surveys or between the IP-IM and IP-RN. In line with expectations, IP respondents completed the survey faster than CAPI and mail respondents.

**Table 8:** Completion Time (Minutes) and Perceived Consequentiality across Survey Mode-Frame Pairs

	Survey Mode-Frame Pairs			
	CAPI	Mail	IP-IM	IP-RN
Completion time				
Mean	21.660	21.290	15.769	15.715
Standard deviation	6.205	9.486	8.277	8.141
Completion time comparison: *t*-test statistic (*P* value)				
Mail	1.007 (0.314)	N/A	—	
IP-IM	17.990 (<0.001)	13.418 (<0.001)	N/A	
IP-RN	18.411 (<0.001)	13.670 (<0.001)	0.148 (0.882)	N/A
Perceived consequentiality, No. (%)				
Strongly disagree	43 (4.10)	44 (4.19)	30 (3.00)	30 (3.00)
Disagree	183 (17.45)	139 (13.24)	134 (13.40)	105 (10.50)
Uncertain	280 (26.69)	386 (36.76)	351 (35.10)	316 (31.60)
Agree	445 (42.42)	389 (31.05)	383 (38.30)	449 (44.90)
Strongly agree	98 (9.34)	92 (8.76)	102 (10.20)	100 (10.00)
Perceived consequentiality comparison (χ^2^ test, *P* value)				
Mail	0.043	N/A	—	
IP-IM	0.121	0.839	N/A	
IP-RN	0.002	<0.001	0.029	N/A

CAPI, computer-assisted personal interviews; IP-IM, Internet panel Ipsos-MORI; IP-RN, Internet panel ResearchNow; mail, postal.

Perceived consequentiality was significantly different across mode-frame pairs; CAPI and IP-RN respondents were more likely than mail respondents to agree or strongly agree that their responses would change how services are provided. However, our hypothesis that CAPI respondents would have the highest perceived consequentiality was not supported; perceived consequentiality was highest for IP-RN respondents.

#### Stated attribute nonattendance

Respondents not engaged with the CE task may use simple rules and shortcuts (heuristics) to make choices (e.g., ignoring attributes). This attribute nonattendance (ANA) behavior has been measured using debriefing questions.^25^ Most CEs aim to elicit respondents’ WTP; therefore, attendance to the cost attribute is of particular interest. We asked respondents whether they considered all attributes and, if not, which attributes they did not consider. We compared ANA across mode-frame pairs using a binomial test. No previous studies have compared stated ANA across modes or frames. If ANA is due to low engagement in the task, then we would expect the CAPI to have lower reported ANA because interviewers are trained to engage respondents in the task.

Table 9 reports the results of 2 comparisons across mode-frame pairs: the proportion of full attribute attendance and, conditional on not attending to all attributes, the proportion of respondents who ignored the cost attribute. The proportions reporting that they attended to all attributes were statistically significantly different across mode-frame pairs: IPs were more likely to report that they considered all attributes compared with CAPI or mail respondents, respectively. Across mode-frame pairs, there was a significant difference in nonattendance to the cost attribute; about 11% of IP respondents reported not attending to the cost attribute compared with 24% of CAPI and 20% of mail respondents. These findings are contrary to our a priori expectations.

**Table 9:** Stated Attribute Nonattendance across Survey Mode-Frame Pairs

	CAPI, No. (%)	Mail, No. (%)	IP- IM, No. (%)	IP- RN, No. (%)	Binomial Test *P* Value
Considered all^a^ Did not consider all	644 (64.4) 356 (35.6)	494 (51.3) 469 (48.7)	709 (74.5) 243 (25.5)	702 (73.5) 253 (26.5)	<0.001
Did not consider^b^:					
Location	112 (31.5)	118 (25.2)	78 (32.1)	69 (27.3)	<0.001
Car parking	186 (52.2)	189 (40.3)	129 (53.1)	124 (49.0)	<0.001
Time	81 (22.8)	54 (11.5)	35 (14.4)	37 (14.6)	<0.001
Who	122 (34.3)	119 (25.4)	81 (33.3)	79 (31.2)	<0.001
Friendly	83 (23.3)	104 (22.2)	71 (29.2)	66 (26.1)	<0.001
Questions	84 (23.6)	98 (20.9)	72 (29.6)	59 (23.3)	<0.001
Understand	76 (21.3)	82 (17.5)	63 (25.9)	50 (19.8)	<0.001
Cost	84 (23.6)	95 (20.3)	25 (10.3)	30 (11.9)	<0.001

There were 89 missing values in the mail survey. CAPI, computer-assisted personal interviews; IP-IM, Internet panel Ipsos-MORI; IP-RN, Internet panel ResearchNow; mail, postal.

a Participants selecting “don’t know/couldn’t say” are not included in this table: CAPI, 49; mail, 70; IP-IM, 48; IP-RN, 45.

b From among respondents reporting attribute nonattendance.

##### Cost per respondent

We compared data collection costs per respondent for each mode-frame pair. We designed a single master survey using word-processing software. A hard copy of that document was posted to the mail sample (with reminders as specified above). The master survey was taken as the basis for scripting the CAPI and IP surveys. The cost per respondent across the mode-frame pairs was £37.50 for the CAPI survey, £18.20 for the mail survey, £8.20 for the IP-IM survey, and £2.50 for IP-RN survey.

## Discussion

We investigated combined survey mode and frame effects in a CE eliciting health care preferences for a health care good: pharmacy services. We compared a commonly used CAPI and mailed survey with the increasingly popular Internet survey. We intentionally confounded survey mode and sample frame^26^; in the CAPI and mail modes, the sample frame was the UK population, whereas for the IP-IM and IP-RN modes, the sample frames were panel members. This confound is inevitable in countries with only volunteer IPs. We used the sample frames researchers normally use. Our results, therefore, represent the (counterfactual) differences researchers might reasonably obtain across modes and the sample frames commonly associated with those modes.

### Response Rates

CAPI had the highest response rate. The mail survey response rate was in line with those reported in other studies.^6^ The IPs had the lowest participation rates. These results are consistent with studies that find Internet participation rates are lower than mail survey response rates.^27^ The low participation rates for the IPs and the finding that about 15% of CAPI and mail respondents do not have Internet access indicate that IP responses may be prone to unobserved sample selection. The response rate to IP-RN was considerably lower than IP-IM. Our pilot study combined an online survey with a PAF sample frame, mailing an invitation to respondents to complete an Internet survey. We received 3 responses to 1000 mailed letters, highlighting the reality that researchers face when considering survey mode and sample frame.

Response rates/participation rates may reflect how good the IPs and PAF are at keeping information up to date. We used resources routinely available to researchers wishing to conduct large-scale surveys of the general public. In the UK, the Royal Mail states that the PAF is “constantly updated” by “making updates to 3,500 records each day” (https://www.royalmail.com/business/services/marketing/data-optimisation/paf). Such a database is recommended for the address-based sampling required for mailed surveys.^28^ When asked about keeping IP membership data up to date, Ipsos Mori commented,

Considerable efforts are taken to maintain engagement of panellists [*sic*]. This would include managing the frequency of invitations, the design of surveys, appropriate incentivisation. It is important to offer panellists the ability to take part in on any type of device, including smartphones. A panellist is free to unsubscribe at any time. Panels are also actively managed and within Ipsos we would purge the panel twice a year to remove those that have not responded to survey invitations.

The response rate for the mail survey, although typical of response rates achieved in CEs,^6^ may have been improved if our contact with the respondents was more personalized.^1^ However, in the UK, there is no suitable postal sample frame that includes names and addresses. Although the electoral register includes names and addresses, it may be used for only a limited set of activities (set by law). Researchers must use the open register, containing a subset of individuals on the electoral register who have not opted out of the open register; about 40% of individuals have opted out of the open register (our own calculation, based on the Office for National Statistics [ONS] estimate that 25,062,982 individuals in England and Wales have opted out [https://www.ons.gov.uk/peoplepopulationandcommunity/elections/electoralregistration/adhocs/008418electorsoptedoutoftheopenregisterforenglandandwalesbylocalauthority2013to2017] and the ONS estimates that 58,381,210 individuals lived in England and Wales in 2017 [https://www.ons.gov.uk/aboutus/transparencyandgovernance/freedomofinformationfoi/ukpopulation2017]).

### Respondent Representativeness

Many mode comparison studies compare respondents’ characteristics across modes but not with the characteristics of the population of interest. Comparisons of respondent characteristics across modes may help explain preference differences. However, these do not address the more important questions of how representative the sample is of the population of interest. Those mode comparison studies that compare their sample with the population of interest do so using a narrower set of socioeconomic characteristics than we did. Using common sampling practice, none of our mode-frame pairs resulted in a fully representative sample. The CAPI performed best in that respondents were representative of the UK population for 3 characteristics (gender, education, and Internet access). The mail survey performed worst; it was not representative of the UK population on any of the 9 representativeness characteristics considered.

As in Mulhern et al.,^3^ we found that quota sampling is no guarantee of representativeness. Our CAPI sample, with a quota applied for age and working status, differed from the UK population in both of these. Our IP samples, with a quota for age, also differed from the UK population. Rowen et al.^4^ found that UK IP respondents were in poorer health than the UK population. We found that respondents to all mode-frame pairs were in poorer health than the UK population. Previous studies have reported that respondents’ perceptions about the personal relevance of a survey can influence response rates.^29–31^ This may explain why people in poorer health are more likely to respond to a survey about the use of health services. Future stated preference surveys should consider this source of nonresponse bias.

Our results regarding the lack of representativeness raise questions regarding how quota sampling is conducted. Quotas for the IPs were filled at the start of the surveys. For both CAPI and IPs, the survey companies used age bands for their quotas broader than those we used. We opted for 5-year age bands that mirror the UK census (e.g., 18–19, 20–24, 25–29, 30–34, 35–39, . . . 85+ years), whereas the survey companies used broader age bands (i.e., 18–24, 25–34, 35–54, 55+ years). This is likely to have been a significant factor in our finding that respondents were not representative (by age) of the UK population. By using the narrower census-based age bands, we wanted to demonstrate one of the effects that researchers might encounter when following the standard practice of commercial survey companies. Analysis of the data using the same age bands used in quotas finds no statistically significant difference for the CAPI, but statistically significant differences still exist for mail and both IPs. Survey companies could sample using narrower age bands; however, it would cost significantly more and is not standard practice in the industry.

### Elicited Values

Our mWTP estimates revealed statistically significant differences across mode-frame pairs. In general, mWTP was highest in the mail survey and lowest for the IPs. Snowball and Willis^32^ found that in-person surveys elicit higher mWTP than self-complete surveys. They suggest this is because in-person respondents are not given enough time to think about their responses to complex CE tasks. Our results do not support this explanation; our mail respondents’ completion times were similar to CAPI respondents. Further, IP respondents completed the surveys faster than both CAPI and mail respondents but reported lower mWTP. An alternative explanation for higher mWTP among CAPI respondents is social desirability bias^33^; respondents may exaggerate their WTP to project a favorable self-image during face-to-face interviews. This, however, would not explain why WTP is highest among mail respondents.

We did not model observable preference heterogeneity. Keane and Wasi^34^ and King et al.^35^ compared econometric models of observable (or scale) heterogeneity captured by interactions between respondents’ characteristics and the CE attributes with mixed logit models of unobservable heterogeneity. They found that respondents’ characteristics explain very little of the variability in preferences found in the mixed logit. The error component logit model we estimated is equivalent to a mixed logit without distributions. Our mean mWTP estimates are unchanged if a mixed logit model with normal distributions for each attribute (except cost) is estimated. Consequently, comparisons of mWTP and tests for differences across the mode-frame pairs are unchanged.

### Data Quality

Our results combine objective measures (response rates, respondent representativeness, and theoretical validity of elicited preferences) with subjective or self-reported measures of data quality (response times, perceived consequentiality, and ANA). Our objective measures may be more reliable. For example, self-reported beliefs about perceived consequentiality are likely to be measured with error.^36^ Perceived consequentiality may be affected by how well respondents remembered, or how much they believed, the information in the study invitation. Mail respondents were significantly less likely to perceive consequentiality; they were also the only group able to refer back to the information sheet when completing the survey (we thank an anonymous reviewer for noting this).

The reliability of respondents’ stated ANA has also been questioned.^37^ We found evidence of inconsistency between the different subjective quality measures, supporting this notion. For example, if ANA measures respondents’ use of simplifying decision heuristics, we would expect respondents attending to fewer attributes to complete the survey faster. However, although CAPI and mail survey respondents were least likely to attend to all attributes, they took longer to complete the surveys. No previous research has investigated how survey mode or frame affects the stated ANA. It is possible that observed differences in the stated ANA were due to the nature of different modes. For example, social desirability may affect the stated ANA if CAPI respondents want to appear altruistic (and therefore not consider cost). Further, IP members received a nominal reward for participation. It might then be argued that IP respondents care more about money than respondents to other modes who give their time freely (we thank an anonymous reviewer for raising these points). Panel membership is also managed by panel providers, and low-quality respondents are removed from panels. This may deter respondents from reporting ANA. Future stated ANA research should consider how mode or frame may affect responses.

### Limitations

Our study has several limitations. Given our aim was to identify expected differences when using different survey modes and the frames commonly associated with them, this limits our ability to isolate the effect of just the modes. Our results represent the differences one might expect to observe between different mode-frame pairs, including the possibility that nonresponse may differ for those pairs. Further, our IP respondents are members of commercially managed access panels that use financial incentives to encourage responses. The use of incentives is standard practice among IP companies and is advocated by experts.^1^ The incentives offered for completing the survey were of low value (actual values were not revealed). It is possible that people volunteering for such panels differ from the general population in ways that we have not controlled for (including those responding to nonincentivized surveys such as the CAPI or mail survey in this study). The incentives offered to Internet panelists may introduce a degree of bias. We compared mWTP, however, income levels were not comparable across mode-frame pairs. We did not include quotas for income in the CAPI or IP surveys. Such quotas are not typical in general population surveys.

## Conclusion

The CAPI is better than, or equivalent to, all other mode-frame pairs on almost all measures, but it is also the most expensive. The mail is the least representative of the population. Our 2 IPs are similar on most measures and are cheaper than other modes. However, we note the extremely low participation rate for IP-RN. While we find many differences, the importance of each of these differences would depend on the context in which the results are used. Researchers need to be aware of these differences when selecting the best survey mode and sampling frame for their study.

## Supplemental Material

Supplemental_material – Supplemental material for Mode and Frame Matter: Assessing the Impact of Survey Mode and Sample Frame in Choice Experiments Supplemental material, Supplemental_material for Mode and Frame Matter: Assessing the Impact of Survey Mode and Sample Frame in Choice Experiments by Verity Watson, Terry Porteous, Tim Bolt and Mandy Ryan in Medical Decision Making
