# Supplementary material for: Mode and Frame Matter: Assessing the Impact of Survey Mode and Sample Frame in Choice Experiments
Source: Med Decis Making. 2019 Sep 15;39(7):827–41. doi: 10.1177/0272989X19871035 (PMC6843610; doi:10.1177/0272989X19871035)
Supplement: Supplemental_material – Supplemental material for Mode and Frame Matter: Assessing the Impact of Survey Mode and Sample Frame in Choice Experiments [file Supplemental_material.pdf]

Appendix Table A1 Beliefs about Medicine Questionnaire

Overuse
If doctors had more time with patients they would prescribe fewer medicines
Doctors use too many medicines
Doctors place too much trust on medicines
Natural remedies are safer than medicines
Harm
Most medicines are addictive
Medicines do more harm than good
People who take medicines should stop their treatment for a while every now and again
Most medicines are poisons
Benefit
Medicines help many people to live better lives
In most cases the benefits of medicines outweigh the risks
In the future medicines will be developed to cure most diseases
Medicines help many people to live longer

The Beliefs about Medicine Questionnaire (BMQ-General) uses a 5-point Likert scale ranging from "strongly agree" to "strongly disagree" with a midpoint "unsure." Permission to use the BMQ-General was sought from its originator, Professor Rob Horne.

Appendix Table A2 Comparison of Age across Survey Mode-Frame Pairs and Census, 2011

	Census, %	CAPI, %	Mail, %	IP-IM, %	IP-RN, %
18 – 19	3.22	2.40	0.29	2.60	1.30
20 – 24	8.65	8.55	1.24	5.90	6.00
25 – 29	8.67	7.78	4.01	5.40	7.30
30 – 34	8.30	8.36	4.01	11.30	11.20
35 – 39	8.44	8.26	4.77	8.90	8.10
40 – 44	9.31	8.93	6.97	8.90	9.60
45 – 49	9.35	8.65	8.68	5.80	9.20
50 – 54	8.24	8.26	9.26	11.70	9.60
55 – 59	7.27	6.34	10.97	10.20	9.40
60 – 64	7.66	6.72	11.74	7.00	7.80
65 – 69	6.07	9.51	13.74	10.20	11.40
70 – 74	4.96	5.76	10.11	7.90	5.70
75 – 79	4.04	5.00	6.49	3.00	1.90
80 – 84	3.01	3.46	4.77	1.00	1.20
85 +	2.81	2.02	2.96	0.20	0.30

The 5-year interval bins are selected to match the data reported by the ONS for the 2011 UK Census. CAPI, computer-assisted personal interviews; IP-IM, internet panel Ipsos-MORI; IP-RN, internet panel ResearchNow; mail, postal.

Appendix Table A3 Comparison of Gender, Education, Self-Assessed Health, Activities of Daily Living, Income, and Internet Access across Survey Mode-Frame Pairs and UK Population

	UK Population, %	CAPI, %	Mail, %	IP-IM, %	IP-RN, %
Gender[1c]	49.1	49.1	40.8	49.2	49.8
Education[2 c]	27.2	29.5	45.7	42.2	43.4
Employment[c]					
employee	52.15	47.21	51.52	52.09	50.21
self-employed	9.48	8.58	8.75	7.62	8.59
unemployed	4.43	6.01	1.8	5.85	5.38
student	9.26	6.55	0.9	5.11	4.97
retired	13.88	19.1	28.51	17.22	18.32
looking after home or family	4.27	8.26	4.6	5.53	4.97
long-term sick or disabled	4.34	3.76	3.59	5.11	6.73
other	2.18	0.54	0.34	1.46	0.83
Self-assessed health[c]					
Very Good/ Good	76.92	71.88	67.78	68.20	69.90
Fair	16.13	20.97	25.51	24.50	21.50
Bad/ Very Bad	6.95	7.15	6.70	7.30	8.60
Activities of daily living[c]					
Yes, limited a lot	10.41	10.30	10.49	8.90	11.10
Yes, limited a little	11.42	14.30	24.84	25.80	23.60
No	78.16	75.41	64.58	65.30	65.30
Income[f]					
Up to £5,199 per year	2.0	7.07	3.11	5.21	5.29
£5,200 to £10,399 per year	6.9	15.10	12.19	8.69	10.92
£10,400 to £15,599 per year	12.9	14.69	13.38	13.33	12.87
£15,600 to £20,799 per year	12.9	11.16	12.90	11.47	12.07
£20,800 to £25,999 per year	10.9	14.01	10.04	12.86	10.57
£26,000 to £31,199 per year	8.9	7.35	8.24	11.59	13.10
£31,200 to £36,399 per year	7.9	7.35	7.17	9.15	7.36
£36,400 to £51,999 per year	15.8	9.66	13.86	13.90	17.13
£52,000 and above per year	21.8	13.61	19.12	13.79	10.69
Internet access[e] *(yes)*	86.8	85.6	83.2	100.0	100.0
Attitude to voting[g]					
It's not really worth voting	16.3	19.64	12.42	12.70	14.10
People should vote only if they care who wins	20.7	19.26	19.32	21.80	22.90
It's everyone's duty to vote	62.0	61.11	68.26	65.50	63.00

Characteristics are reported and compared for respondents who completed the questions and excluding item nonresponse. CAPI, computer-assisted personal interviews; IP-IM, internet panel Ipsos-MORI; IP-RN, internet panel ResearchNow; mail, postal.

[1]Gender is measured as proportion of the UK population/sample who are male. [2]Education is measured as the proportion of the UK population/sample who have at least a higher education qualification. [c]UK population data source is UK census 2011, [f]UK population data source is Family Resources Survey, [e]UK population data source is OECD statistics (https://data.oecd.org/ict/internet-access.htm) based on Eurostat Community survey on ICT usage in households and by individuals on Eurostat Community survey on ICT usage in households and by individuals. [g]UK population data source is UK Social Attitudes Survey.

Appendix Table A4 Comparison of Chronic Illness, Membership of Minor Ailments Scheme, and Beliefs about Medicine Questionnaire across Survey Mode-Frame Pairs

	CAPI, %	Mail, %	IP-IM, %	IP-RN, %
Chronic illness/ *self-reported "Yes"*	29.74	47.46	0	0
Minor ailments scheme				
Yes	4.00	2.00	1.71	2.02
No	91.33	90.59	91.16	92.22
Not Sure	4.67	7.41	7.14	5.67
Beliefs about Medicine Questionnaire[a]				
Overuse	46.90	40.55	42.10	38.10
Harm	18.78	12.12	16.90	11.70
Benefit	91.13	93.49	90.90	91.50

Characteristics are reported and compared for respondents who completed the questions and excluding item nonresponse. CAPI, computer-assisted personal interviews; IP-IM, internet panel Ipsos-MORI; IP-RN, internet panel ResearchNow; mail, postal.

[a]*The table presents results for the three scales included within the Beliefs about Medicine Questionnaire. The percentage of each sample with an average score of 3 or above (out of 5) for that scale is reported.*
